# Cell Cycle Modulation by Marek’s Disease Virus: The Tegument Protein VP22 Triggers S-Phase Arrest and DNA Damage in Proliferating Cells

**DOI:** 10.1371/journal.pone.0100004

**Published:** 2014-06-19

**Authors:** Laëtitia Trapp-Fragnet, Djihad Bencherit, Danièle Chabanne-Vautherot, Yves Le Vern, Sylvie Remy, Elisa Boutet-Robinet, Gladys Mirey, Jean-François Vautherot, Caroline Denesvre

**Affiliations:** 1 INRA, UMR1282 Infectiologie et Santé Publique, Equipe Biologie des Virus Aviaires, Nouzilly, France; 2 INRA, UMR1282 Infectiologie et Santé Publique, Laboratoire de Cytométrie, Nouzilly, France; 3 INRA, UMR 1331, Toxalim, Research Centre in Food Toxicology, Toulouse, France; 4 University of Toulouse, UPS, UMR1331, Toxalim, Toulouse, France; Queen’s University, Canada

## Abstract

Marek’s disease is one of the most common viral diseases of poultry affecting chicken flocks worldwide. The disease is caused by an alphaherpesvirus, the Marek’s disease virus (MDV), and is characterized by the rapid onset of multifocal aggressive T-cell lymphoma in the chicken host. Although several viral oncogenes have been identified, the detailed mechanisms underlying MDV-induced lymphomagenesis are still poorly understood. Many viruses modulate cell cycle progression to enhance their replication and persistence in the host cell, in the case of some oncogenic viruses ultimately leading to cellular transformation and oncogenesis. In the present study, we found that MDV, like other viruses, is able to subvert the cell cycle progression by triggering the proliferation of low proliferating chicken cells and a subsequent delay of the cell cycle progression into S-phase. We further identified the tegument protein VP22 (pUL49) as a major MDV-encoded cell cycle regulator, as its vector-driven overexpression in cells lead to a dramatic cell cycle arrest in S-phase. This striking functional feature of VP22 appears to depend on its ability to associate with histones in the nucleus. Finally, we established that VP22 expression triggers the induction of massive and severe DNA damages in cells, which might cause the observed intra S-phase arrest. Taken together, our results provide the first evidence for a hitherto unknown function of the VP22 tegument protein in herpesviral reprogramming of the cell cycle of the host cell and its potential implication in the generation of DNA damages.

## Introduction

Gallid herpesvirus 2 (GaHV-2), more frequently referred to as Marek’s disease virus (MDV), is an alphaherpesvirus (type species of the genus Mardivirus) and the causative agent of a highly infectious lymphoproliferative disease termed Marek’s disease (MD) affecting many birds in the *Phasianidae* family. Despite global vaccination campaigns that are effective to prevent disease development, MDV field strains continue to spread in poultry and appear to evolve towards increased virulence. The dissemination of MDV in poultry is mediated by infectious viral particles associated with dander and feather debris [1], [2]. With the exception of the feather follicle epithelium, the site where free infectious viral particles are shed, the virus remains strictly cell-associated and progression of the infection is restricted to viral cell-to cell spread [3]. The MDV particle is composed of a 180-kbp double-strand DNA genome packaged in an icosaedric capsid surrounded by a tegument layer, which insures the morphological and functional continuity between the capsid and the host cell derived viral envelope. By homology with other alphaherpesviruses, a number of viral proteins composing the tegument have been identified, including a major tegument protein, VP22 (pUL49), various trans-activators and two protein kinases (pUL13 and pUS3). The UL49-encoded VP22 protein is abundantly expressed in infected cells and is essential for MDV replication [4], [5], [6]. VP22 is a specific tegument protein of alphaherpesviruses and conserved among this subfamily. To date, the absolute requirement of the UL49 gene for viral replication was initially demonstrated for MDV [5] and afterwards for Varicella Zoster virus (VZV) [7]. The deletion of VP22 in other alphaherpesviruses including Herpes Simplex virus 1 (HSV-1), Pseudorabies virus (PRV), Bovine herpesvirus 1 (BoV-1) still allows viral replication, even though viral spread is reduced in some cell types [8], [9], [10], [11], [12]. While its role in virus infection remains unclear, it was demonstrated for HSV-1 that VP22 interacts with and recruits various viral proteins, such as the trans-activators ICP0, ICP4 and viral glycoproteins composing the infectious virions [9], [10], [13]. Furthermore, VP22 was shown to interact with cellular proteins involved in the organization of microtubules and nucleosome assembly [14], [15]. The VP22 protein encoded by MDV shares common functional features with VP22 encoded by other alphaherpesviruses [5], [16]. It was previously shown that MDV-VP22 shows both a cytoplasmic and nuclear location in infected cells and accumulates in the nucleus upon overexpression in cells [4]. Moreover, MDV-VP22 exhibits a strong affinity to DNA, especially heterochromatin, and to microtubules [4], [17]. We previously demonstrated the role of VP22 in MDV cell-to-cell spread, which could explain the necessity of VP22 in MDV replication [16], [18]. It was recently shown that recombinant MDV viruses expressing VP22 with a C or N-terminal GFP-tag are highly attenuated *in vivo* suggesting that VP22 might play a role in MDV-induced lymphomagenesis [6], [19]. However, the precise role of VP22 in MDV replication and MD pathogenesis remains unclear. Notably, the functional significance of the VP22 nuclear distribution is still unknown, even if previous reports on VP22 encoded by alphaherpesviruses evoke a possible regulatory function of VP22 within nuclei [17], [20], [21], [22].

Virus infection frequently results in the disturbance of key cellular processes within the host cell. The subversion of cell cycle pathways is a well-established mechanism by which viruses create the most suitable environment for their replication. Especially, the induction of S-phase is either mandatory or at least advantageous for lytic replication of a number of viruses. The eminent role of cellular factors from the DNA synthesis machinery in viral replication was demonstrated for viruses from different families such as the Flaviviridae, Retroviridae, Parvoviridae, and Polyomaviridae [23], [24], [25], [26], [27], [28]. In contrast, herpesviruses encode their own DNA polymerase and accessory proteins, and thus theoretically do not require an S-phase environment to support their replication (reviewed in [29], [30]). Nevertheless, several studies have demonstrated the importance of the S-phase in the life cycle of VZV and Epstein-Barr virus (EBV) [31], [32]. For EBV, S-phase cyclin-dependent kinase activity is essential for the expression of immediate early and early viral proteins and is thus required for viral replication [31]. *Vice versa*, EBV lytic replication is able to provide a S-phase-like cellular environment by modulating DNA damage pathways [33]. The impact of the S-phase environment on the viral life cycle is not restricted to lytic viral replication but is also involved in the episomal genome maintenance during viral latency or reactivation processes, as was recently shown for EBV and the Kaposi’s Sarcoma-associated herpesvirus (KSHV) [34], [35]. Strikingly, infections with oncogenic viruses (e.g., SV40, HPV, HTLV-1, EBV) are often associated with S-phase deregulation and genomic instability, preferentially occurring during this critical phase of the cell cycle [24], [27], [33], [36].

In relation with cell cycle delay, DNA damage signaling is often triggered upon viral infections (reviewed in [37], [38]). Particularly, DNA damage response (DDR) pathways are preferential targets of herpesviruses, including HSV-1, EBV, KSHV, human cytomegalovirus and murine gamma-herpesvirus 68 [33], [39], [40], [41], [42], [43]. The role of DDR in herpesviruses life cycle is complex. On the one hand, recent evidence suggests that DDR acts as an efficient antiviral response [44]. On the other hand, DDR modulation can be beneficial for herpesviruses by facilitating viral replication, viral genome processing or latency establishment [33], [34], [40], [42], [45]. Moreover, during the course of cellular infections with large DNA tumour viruses, such as human papillomaviruses (HPV) or gammaherpesviruses (e.g. EBV and KSHV), the generation of DNA damage and/or activation of DDR were found to be associated with genomic instability which in turns can participate to virus-induced tumorigenesis (reviewed in [46], [47], [48]).

In the present study, we set out to elucidate an important aspect of MDV-host cell interaction by analyzing the impact of MDV and virus-encoded proteins on the regulation of the cell cycle. We demonstrate that MDV lytic infection activates the proliferation of chicken primary skin cells concomitant with a delay in S-phase. By studying the effects of transient vector-driven overexpression in a proliferating chicken cell line, we identified the VP22 tegument protein as a potent cell cycle modulator encoded by MDV. A comparative experimental approach employing VP22 variants with a C- or N-terminal eGFP-tag allowed us to show that an unmodified C-terminus of VP22 is required to elicit the observed S-phase arrest. Moreover, the cell cycle regulating activity of VP22 relies on its ability to be associated with chromatin in the nucleus. In order to define the mechanisms underlying the drastic S-phase arrest observed in VP22 expressing cells, we investigated the impact of VP22 expression on DNA integrity. Strikingly, we found that the DNA of cells expressing this viral protein showed significant DNA damage, as was assessed by comet assay. Together, these data provide new insights into the interaction of MDV with the host cell during lytic replication and pinpoint to a novel powerful function of VP22 that may help to better understand the pre-eminent role of VP22 in MDV replication and more generally in the life cycle of the virus.

## Materials and Methods

### Cell Culture and Viruses

Chicken Embryo Skin Cells (CESC) were prepared from 12-days-old chicken embryos (LD1 Brown Leghorn chicken strain) and maintained in culture as previously described [4]. This procedure was carried out in strict compliance with the French legislation for animal experiments and ethics stating that the use of embryos from oviparous species before the last third of their normal development (i.e. before day 14 for chicken embryos) is not submitted to regulation (Art. R.214-88). Thus, the preparation of CESC from 12-days-old chicken embryos does not require the permission of governmental or local authorities. Embryos were sacrificed by opening the eggshell, cervical dislocation and immersion in William’s Medium E (Lonza) supplemented with collagenase as described by Dorange *et al*., 2000. The chicken hepatocellular carcinoma cell line LMH was cultured on gelatin-coated flasks in William’s Medium E (Lonza) supplemented with 2 mM glutamine and 10% fetal bovine serum (FBS) at 37°C in a 5% CO2 atmosphere. As positive controls for DNA damage analyses, LMH cells were treated for 24 h with 1.5 µM etoposide, a topoisomerase IIα inhibitor potent inducer of DNA double strand breaks.

Recombinant viruses were generated from the avirulent MDV-BAC20 strain cloned as bacterial artificial chromosome (BAC) [49]. The recEGFPVP22 recombinant virus harboring the UL49 gene fused at its 5′ end with the eGFP gene was previously described [16].

Parental BAC20 and recEGFPVP22 viruses were produced after transfection of BAC-DNA into CESC as previously reported [16]. Infections were performed by co-culture of 7×10^6^ fresh CESC in a 100-mm diameter plate with infected cells at a ratio of 10^4^ PFU/plate.

### Plasmids

The pcDNA3-UL49 and pcDNA3-UL48 plasmids encoding the wild-type (wt) VP22 and VP16 tegument proteins of the RB-1B oncogenic MDV-RB-1B strain, have been previously described [4]. Two plasmids harboring the VP22 protein cloned in frame with the enhanced green fluorescent protein (eGFP) were used: (i) the peGFP-UL49, encoding a VP22 tagged with eGFP at its N-terminal extremity [18] and (ii) the pUL49-eGFP in which VP22 is tagged with eGFP at its C-terminus. The latter construct was generated by PCR amplification of the UL49-eGFP fragment from the purified rUL49-eGFP BAC-DNA kindly provided by B. Kaufer (Institut für Virologie, Freie Universität Berlin, Germany) [19]. The primer pairs used for amplification were UL49FCLBamHI/eGFPendNotI (Table 1). The PCR product was inserted into the PCR2.1 TOPO TA cloning vector (Invitrogen) and the BamHI/NotI fragment was then sub-cloned into the peGFP-N1 vector (BD Biosciences, Clontech) where the internal eGFP cassette was previously removed by BglII/NotI enzymatic cleavage. The pGE109 plasmid harbouring the UL49 gene encoded by HSV-1 was kindly provided by G. Elliott [50]. The HSV1-UL49 gene was cloned in frame with eGFP at the Bgl II site in the peGFP-C1 vector (BD Biosciences, Clontech). The VZV-ORF63 encoding the VZV orthologue of UL49 was amplified from pcDNA63wt (kindly provided by C. Sadzot-Delvaux) with the primer pair 5FUL49VZVXhoI/3RUL49VZVBamHI (Table 1) [51]. The PCR product was T/A-cloned into the pGEMT-easy cloning vector (Promega) and subsequently subcloned in fusion with eGFP in the peGFP-C1 vector at the Xho I and Bam HI sites.

**Table 1:** Primers used for the cloning of the genes of interest in eukaryotic expression vectors.

Primer names	Primer sequences
UL49FCLBamHI(1)	5′GG**GGATCC**CATGGGGGATTCTGAAAGGCGG3’
eGFPendNotI(2)	5′AGGCGGCCGCTTACTTGTACAGCTCGTCCATG3’
5FUL49VZV XhoI(3)	5′GG**CTCGAG**CTTTGGCATCTTCCGACGGTGA3’
3RUL49VZVBamHI(1)	5′GG**GGATCC**CTATTTTCGCGTATCAGTTC3’
UL37F	5′AGGCCTATGTCTGCCGTAACGACCGA3’
UL37R	5′CTGCAGTTATGCATTATCACCGTTTG3’
UL13F	5′CCAGATCTATGGATACTGAATCAAAAAAC3’
UL13R	5′CCGAATTCCTAGTTCCATAACAACAAATC3’
UL54F	5′AGGCCTATGTCTGTAGATGCATTCTCTC3’
UL54R	5′AGGCCTCTAGATTACATACCAAACAGAGTATTGCAG3’
US3F	5′CCAGATCTATGTCTTCGAGTCCGGAGGC3’
US3R	5′CCGAATTCTTACATATGAGCGGCAGTTATC3’

(1) BamHI restriction site is indicated in bold.

(2) NotI restriction site is underlined.

(3) XhoI restriction site is indicated in bold underlined.

The genes encoding UL37 (pUL37), UL54 (encoding the ICP27 trans-activator), and the two viral kinases UL13 (pUL13) and US3 (pUS3) were amplified from RB-1B genomic DNA with the primer pairs UL37F/UL37R; UL13F/UL13R; UL54F/UL54R, and US3F/US3R, respectively (Table 1). Amplification products were inserted into the pGEMT-easy cloning vector (Promega). The UL37, UL13, and US3 genes were sub-cloned under control of the cytomegalovirus immediate early promoter into the pcDNA3.1 vector (Invitrogen) at the NotI site, and the UL54 gene was cloned into the pcDNA3.1 vector at the EcoRV site. All intermediate and final constructs were verified by sequencing (Eurofins, MWG Operon).

### Transient Expression

The different eukaryotic expression vectors were transfected into CESC or LMH by using Lipofectamine 2000, according to the manufacturer’s instructions (Invitrogen). Briefly, cells at 80% of confluency plated on 60-mm dishes were rinsed twice with OptiMEM (Fischer Scientific) and were transfected with 5 µg of the plasmid of interest. After 6 h of incubation at 37°C, the transfection mix was removed and serum complemented fresh medium was added. Cells were harvested 24 h or 48 h after transfection for further analysis. Each transfection was performed in triplicate.

### Cell Cycle Analysis

At the time points indicated, 1.10^6^ vector-transfected or infected cells were trypsinized and washed twice in phosphate-buffered saline (PBS) prior to fixation with 70% ethanol at 4°C for 24 h. Cells were then washed twice in cold PBS and incubated in PBS containing 500 µg/ml Ribonuclease A (Sigma-Aldrich) at 37°C for 1 h. After filtration through a 30-µm pore size membrane, cells were stained with 10 µg/ml propidium iodide (Invitrogen) for 15 min in the dark. Flow cytometry analysis was performed using a MoFlo high-speed cell sorter (Beckman Coulter, Fort Collins, CO, USA) equipped with a solid-state laser operating at 488 nm and 100 mW. Cellular DNA content was analyzed with a 740 nm long-pass filter. Doublets were discarded on the basis of combination of pulse width and area/peak fluorescence. eGFP autofluorescence was detected with a 530/40 nm band-pass filter and the cell cycle distribution was specifically analyzed for eGFP-positive versus eGFP-negative cells. Cell cycle profiles were analyzed with the MultiCycle AV software (Phoenix Flow Systems, California, USA).

### Reverse Transcription-Polymerase Chain Reaction (RT-PCR) and Real Time Quantitative RT-PCR (qRT-PCR)

Total RNA was extracted from 10^6^ cells with Trizol according to the manufacturer’s instructions (Sigma-Aldrich). RNAs were treated with RNAse-free RQ1 DNAse (Promega, France) and RNA concentration was measured with a NanoDrop spectrophotometer. One µg of each total RNA preparation was reverse transcribed using 100 µg/mL oligo(dT) primers (Promega) and M-MLV reverse transcriptase according to the manufacturer’s recommendations (Promega).

The expression of the different cellular genes involved in cell cycle regulation was analyzed by qPCR. Amplification of the cDNA by qPCR (CFX96 Touch Real-Time PCR Detection System; Bio-Rad) was performed in triplicate, using 200 ng of cDNA, 7.5 µl 2×iQ Supermix SYBR green (Bio-Rad), 1 µl ultrapure water (Sigma-Aldrich) and 0.75 µl of each specific primer (10 µM) selected according to the EST data deposited in Genbank (described in Table 2). The PCR program consisted of a 5 min activation step at 95°C, followed by 39 cycles of 95°C for 10 s and 60°C for 10 s. Expression of the chicken glyceraldehyde phosphate dehydrogenase (GAPDH) was used for the normalization of all target gene mRNAs to enable cross-comparisons among the samples. The relative changes in gene expression were determined by the 2(−ΔΔCT) method.

**Table 2:** List of forward (For) and reverse (Rev) primers used for (q)RT-PCR.

Target gene		Primers sequences	Accesion number gene
Cyclin A	For	5′CAAGCTCCAGAATGAAACTC3’	GenBank X72892
	Rev	5′GATGTAGACGAACTCTGCTA3’	
Cyclin B	For	5′GTTCTGTCTCCTGTCCCTAT3’	GenBank X62531
	Rev	5′AGCTCAAGCTGTCTCAGATA3’	
Cyclin D	For	5′GCATTTACACCGACAACTCC3’	GenBank NM_205381
	Rev	5′GCATGTTTACGGATGATCTG3’	
Cyclin E	For	5′TCACCGCTACCAATTCTGGG3’	ENSGALT00000007159
	Rev	5′ACTTCACAAACCTCCATTAG3’	
Cdk1	For	5′GTAGTGACACTGTGGTACAG3’	GenBank NM_205314
	Rev	5′CTGAAGATTCTGAAGAGCTG3’	
Cdk6	For	5′ATGTTGATCAGCTAGGAAAA3’	GenBank NM_001007892
	Rev	5′CGATTTAAGAAGCAAGTCTT3’	
pRb	For	5′GATGTGTTCCATGTATGGCA3’	GenBank NM_204419
	Rev	5′TGAACACTAAGTTGTAGAAG3’	
p53	For	5′CAGCCAAATCGGTCACCTGC3’	Genbank NM_205264
	Rev	5′CCGCACCACTTCGGCCACGT3’	
c-Myc	For	5′CCGAGGACATCTGGAAGAAGTT3’	GenBank J00889.1
	Rev	5′TCGCAGATGAAGCTCTGGTTGA3'	
E2F1	For	5′CGGTGAAGCGGAAGCTGAAC3’	GenBank NM_205219
	Rev	5′GCTCCAGGAAGCGCTTGGTG3’	
ICP4	For	5′TTTCTAGCAAGGAGCGACGC3’	GenBank NC_002229.3
	Rev	5′CTGACTTGCGCTTACGGGAA3’	
UL13	For	5′CTCGGCAAAGCAGTTGTGTTTC3’	Genbank EF523390
	Rev	5′GTCAGACTAAAATCACCAATTAC3’	
US3	For	5′CGCCTGAACTGCTTGCACTTG3’	Genbank EF523390
	Rev	5′TGGATCTCAGCTGAGAACCTG3’	
UL49	For	5′GGAAGACGTTTCGTCTACCAC3’	Genbank EF523390
	Rev	5′CATACGCTGATTAAATGCCACTG3’	
UL37	For	5′GCGCATTGGGTTCGAAAGAAC3’	Genbank EF523390
	Rev	5′CGTTGCGCAACTTATCAGCCG3’	
UL54	For	5′CTCGCGAGTCCGATGACATG3’	Genbank EF523390
	Rev	5′GTTCCTGCGTACACGGTGGC3’	
UL48	For	5′CGAAGCATCTCAAATGGGACG3’	Genbank EF523390
	Rev	5′CTGAGCCCGGAGTTCAGGAG3’	
GAPDH	For	5′TGATGATATCAAGAGGGTAGTGAAG3’	GenBank K01458
	Rev	5′TCCTTGGATGCCATGTGGACCAT3’	

The expression of MDV genes (ICP4, UL13, US3, UL49, UL37, UL54 and UL48) was assessed by RT-PCR performed with 100 ng of the synthesized cDNA prepared from LMH or CESC cells transfected with the corresponding expression vector and 10 µM of specific primers (Table 2). The GAPDH gene was used as internal control. Specific PCR products were resolved by agarose (2%) gel electrophoresis.

### Immunofluorescence Microscopy

At 24 h or 48 h post-transfection, cells grown on glass coverslips were fixed with 4% paraformaldehyde (PFA) for 20 min at room temperature (RT), permeabilized with 0.5% Triton X-100 for 5 min at RT and blocked with PBS, 0.1% Triton X-100, 2% Bovine Serum Albumin (BSA). Immunostainings were performed with monoclonal antibodies directed against phospho-histone H2AX (Ser139) (Millipore; clone JBW301) and tubulin (Sigma-Aldrich; catalog number T9026) at a dilution of 1∶250 and 1∶500, respectively. Goat anti-mouse IgG Alexa-Fluor 594 secondary antibody (Invitrogen) was used at 1∶2000. Cell nuclei were counterstained with Hoechst 33342 dye (Invitrogen). Cells were observed under an Axiovert 200 M inverted epifluorescence microscope equipped with the Apotome imaging system (Zeiss). Images were captured with an Axiocam MRm camera and analyzed by using the Axiovision software (Zeiss). To determine the cellular distribution of eGFP-tagged proteins, a minimum of 100 transfected cells were observed and the results were presented as percentage reflecting the nuclear and/or cytoplasmic distribution of the protein.

### Cell Sorting

RecEGFPVP22-infected cells or LMH cells transfected with peGFP vectors were trypsinized 24 h post-transfection and filtered on a 30-µm-pore-size membrane. eGFP positive and negative cells were sorted with a MoFlo (Beckman Coulter, Fort Collins, CO, USA) high-speed cell sorter equipped with a solid-state laser operating at 488 nm and 100 mW. Damaged cells and debris were eliminated on the basis of morphological criteria. eGFP fluorescence was analyzed with a 530/40 nm band-pass filter. The sorting speed was around 15,000 cells/s and cells were collected in appropriate media supplemented with 10% of FBS.

### Alkaline Comet Assay

LMH cells transfected with the peGFP, peGFP-UL49 or pUL49-eGFP were harvested 24 h post-transfection and eGFP positive and negative cell were sorted by flow cytometry. After sorting, 2.10^5^ cells were used to prepare 3 slides for comet assays, realized as previously described with minor modifications [52]. Electrophoresis was performed at 0.7 volts/cm for 26 min with the Sub-cell GT agarose gel electrophoresis system (Bio-Rad). DNA was then stained with a 20 µg/ml ethidium bromide solution and slides were observed using the Axiovert 200 M inverted epifluorescence microscope (Zeiss). Images were captured with an Axiocam MRm camera (Zeiss) and comets were analyzed with the CometScore software version 1.5 (TriTek). The Tail Extend Moment (TEM) was calculated on the basis of the comet tail length and the relative proportion of DNA contained in the tail. Experiments were carried out 3 times and for each experiment, a minimum of 50 comets was analyzed on each of the 3 slides. Results are presented as the mean (±SD) of the TEM calculated for each condition or as a distribution of the comets with respect to their respective TEM value.

### High Salt Extraction of Histones

Salt extraction of histones from chromatin was performed as previously described [53]. Briefly, 1.10^7^ cells were resuspended in 1 ml extraction buffer (340 mM Sucrose, 10 mM Hepes pH 7.9, 10 mM KCl, 1.5 mM MgCl_2_, 10% glycerol) containing 0.2% Igepal (Sigma-Aldrich) and 1X protease inhibitors (Complete Mini EDTA free, Roche). After incubation on ice for 10 min, the soluble fraction was separated from the nuclei by centrifugation at 6,500×g for 5 min. Nuclei were resuspended in 1 ml no-salt lysis buffer (3 mM EDTA, 0.2 mM). After incubation at 4°C for 30 min, the chromatin was pelleted by centrifugation at 6,500×g for 5 min, and incubated in 500 µl of high-salt solubilization buffer (50 mM Tris-HCl pH 8.0, 2.5 M NaCl and 0,05% NP40) for 30 min at 4°C. Nuclear debris were pelleted by centrifugation at 16,000×g for 10 min and the supernatant containing the histones fraction was collected. The proteins included in this fraction were separated in a 10% SDS-PAGE gel and revealed with colloidal coomassie blue staining (Sigma-Aldrich). Detection of VP22 was accomplished by immunoblotting using the monoclonal anti-VP22 antibody (L13a, [4]) diluted 1∶1000 and an anti-mouse IgG conjugated to horseradish peroxidase (HRP) (Sigma-Aldrich). Specific protein signals were detected with the Pierce ECL2 Western Blotting Substrate (Thermo Scientific) and the Fusion-FX7 imaging system (Vilber Lourmat). Quantification was carried out using the Bio-profil 1D++ software (ChemiSmart 5000).

### Statistical Analysis

All graphs and statistics were performed using the GraphPad Prism software version 5.02 (San Diego, USA). Data are presented as means and standard deviations (±SD). Significant differences were determined using Student’s *t*-test. *P* values <0.05 were considered statistically significant.

## Results

### MDV Infection Delays Cell Cycle Progression in S-phase

**Figure 1:**
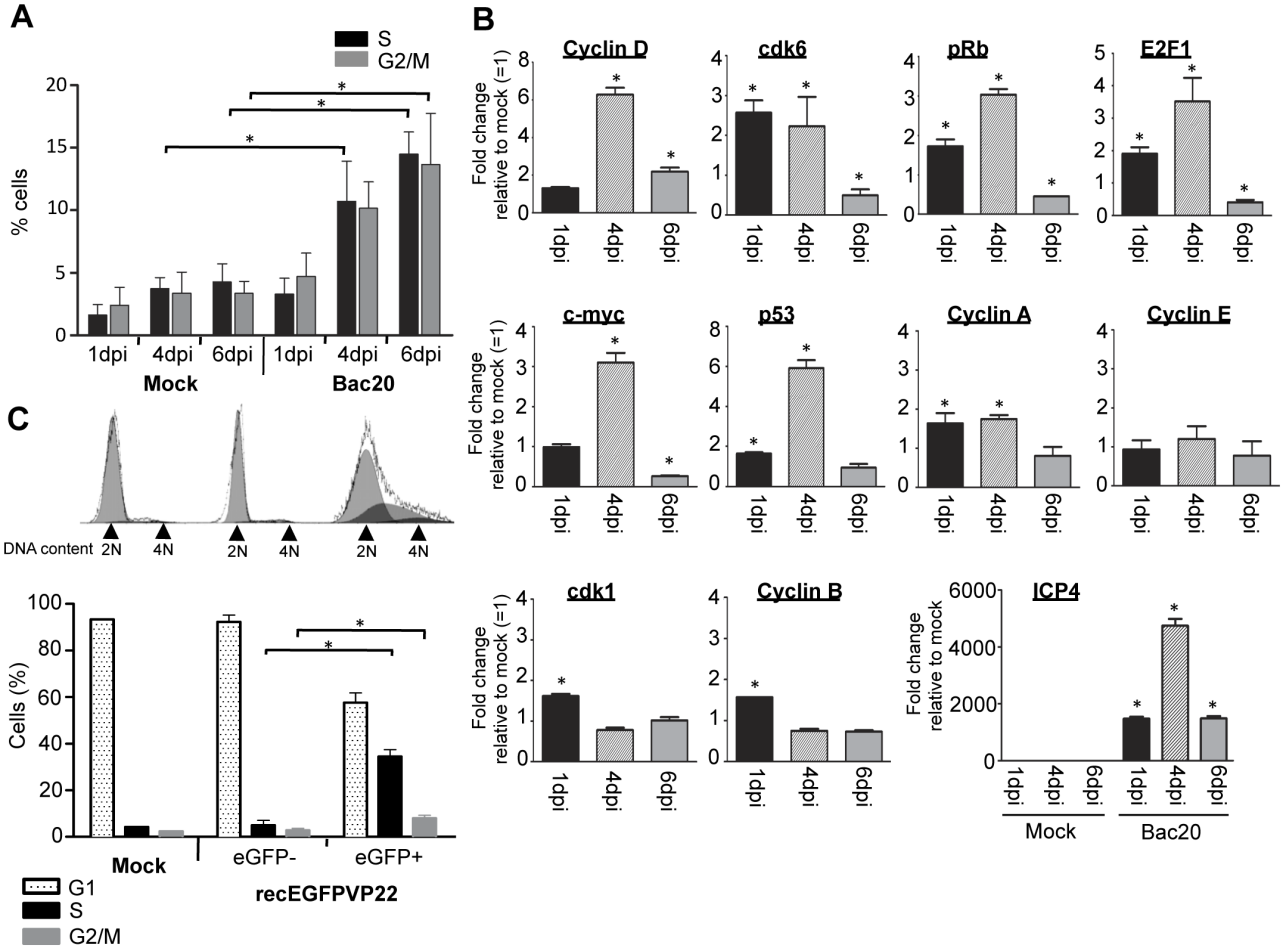
Cell cycle regulation during MDV infection. (A) Cell cycle analysis of CESC infected with the BAC20 virus. Mock-infected or infected CESC were harvested 1, 4, and 6 days pi and the cellular DNA content was analyzed by flow cytometry after staining with propidium iodide. The percentage of cells in S and G2/M phases is represented as bars. *p<0.05. (B) mRNA expression of cell cycle regulators during MDV infection. RNAs were extracted from mock-infected and BAC20-infected cells at indicated time points. The mRNA expression levels of cellular key cell cycle regulators were detected by qRT-PCR using specific primers given in Table 2. To assess lytic viral replication, the expression of the ICP4 gene encoded by MDV was analyzed in parallel by qRT-PCR. Expression of GAPDH mRNA was used for the normalization of mRNA expression levels of all target genes and the relative changes in gene expression were determined by the 2(−ΔΔCT) method. Results are presented as histograms showing the fold-change of expression of the target gene in infected cells relative to its expression in mock-infected cells (Mock = 1). *p<0.05. (C) Cell cycle regulation in MDV infected cells. CESC were mock-infected or infected with the recEGFPVP22 virus. Six days post-infection, cells were trypsinized, fixed, and the DNA was stained with propidium iodide. DNA content was analyzed by flow cytometry in late infected cells (eGFP+) and non-infected cells (eGFP−). Single parameter histograms are shown, and the results corresponding to the percentage of cells in G1, S, and G2/M phases of 3 independent experiments is presented as mean (±SD). *p<0.05.

In order to analyze the influence of MDV infection on cell cycle progression, chicken embryonic skin cells (CESC) were infected with the parental BAC20 virus. At days 1, 4, and 6 post-infection (pi), mock- and virus-infected cells were fixed in ethanol, DNA was stained with propidium iodide and DNA content was analyzed by flow cytometry (Fig. 1A). While no significant difference in the cell cycle progression was observed in the early steps of infection (1 dpi), at day 4 pi the cell population in S-phase in BAC20-infected cells was about 3-fold higher than in mock-infected cells. At day 6 pi, the proportion of cells in S-phase as well as in the G2-phase remained 3-fold higher in BAC20-infected cells, suggesting that MDV infection activates cell cycle progression of CESC that normally exhibit a low proliferating rate (3 to 4% of cells in S-phase) and that MDV may delay the cell cycle in S-phase. In order to ascertain viral replication, mRNA expression of the early ICP4 viral gene was followed by qRT-PCR (Fig. 1B lower panel). To confirm the activation of the cell cycle progression assessed by DNA content analysis, and to define the molecular mechanisms of this process upon infection, we examined the expression of key factors involved in cell cycle regulation including cyclins and cyclin dependent kinases (cdk). CESC were mock-infected or infected with BAC20, and qRT-PCR analyzes were performed on total mRNAs extracted at 1, 4, and 6 days pi (Fig. 1B). At 4 dpi, BAC20-infected cells showed an increase of the mRNA expression of cyclin D (of about 6 fold), cdk6 (2.3-fold), pRb (3-fold), E2F1 (3.5-fold) and c-myc (3.1-fold) compared to mock-infected control cells. A slight up-regulation of cyclin A (1.7-fold), cyclin B (1.5-fold) and cdk1 (1.6-fold) mRNA expression was also detected at 1 dpi in infected cells compared to non-infected cells, while the level of cyclin E mRNA expression was comparable to that in mock-infected cells. These observations are in good agreement with the DNA content analyses showing an activation of the proliferative capacities of infected CESC, since cell cycle progression markers, especially cellular factors involved in the progression into G1 and S-phases (cyclin D, cyclin A, cdk6, pRb, c-Myc, and E2F1) were up-regulated during MDV infection. Interestingly, analysis of the mRNA expression pattern of p53, a protein crucially involved in cell cycle checkpoints and DNA damage pathways, revealed a strong up-regulation (of about 5.9 fold) of its expression at 4 dpi. Of note, we also observed a down-regulation of the mRNA expression of cdk6, pRb, E2F1 and c-myc at 6 dpi that reflect the non-progression of the cell cycle in G1/S phase.

To specifically determine the regulation of the cell cycle in infected cells and to preclude problems associated to asynchronous infection and moderate infectivity titers, CESC infected with the recEGFPVP22 virus, an MDV recombinant virus expressing an eGFP-VP22 fusion protein, were sorted by flow cytometry [18]. Infected cells monolayers were harvested at 6 days pi, and the DNA content in non-infected (eGFP-negative) and infected cells (eGFP-positive) was analyzed by flow cytometry. Using this approach, we could observe that 34.4% of the eGFP-positive cells were delayed in S-phase (Fig. 1C), while in the eGFP-negative population the percentage of cells in S-phase was equivalent to that in mock-infected cells (up to 5%). In addition, a slight increase of cells in G2-phase was detected in infected cells (8% compared to 2.8% in eGFP-negative cells or mock-infected cells). All together, these data clearly demonstrate that lytic MDV infection drives primary avian cells into an active proliferating state. Furthermore, the significant increase of MDV-infected cells accumulating in S-phase is also indicative of a virus-mediated delay in S-phase progression.

### VP22 is a Major Cell Cycle Regulator

**Figure 2:**
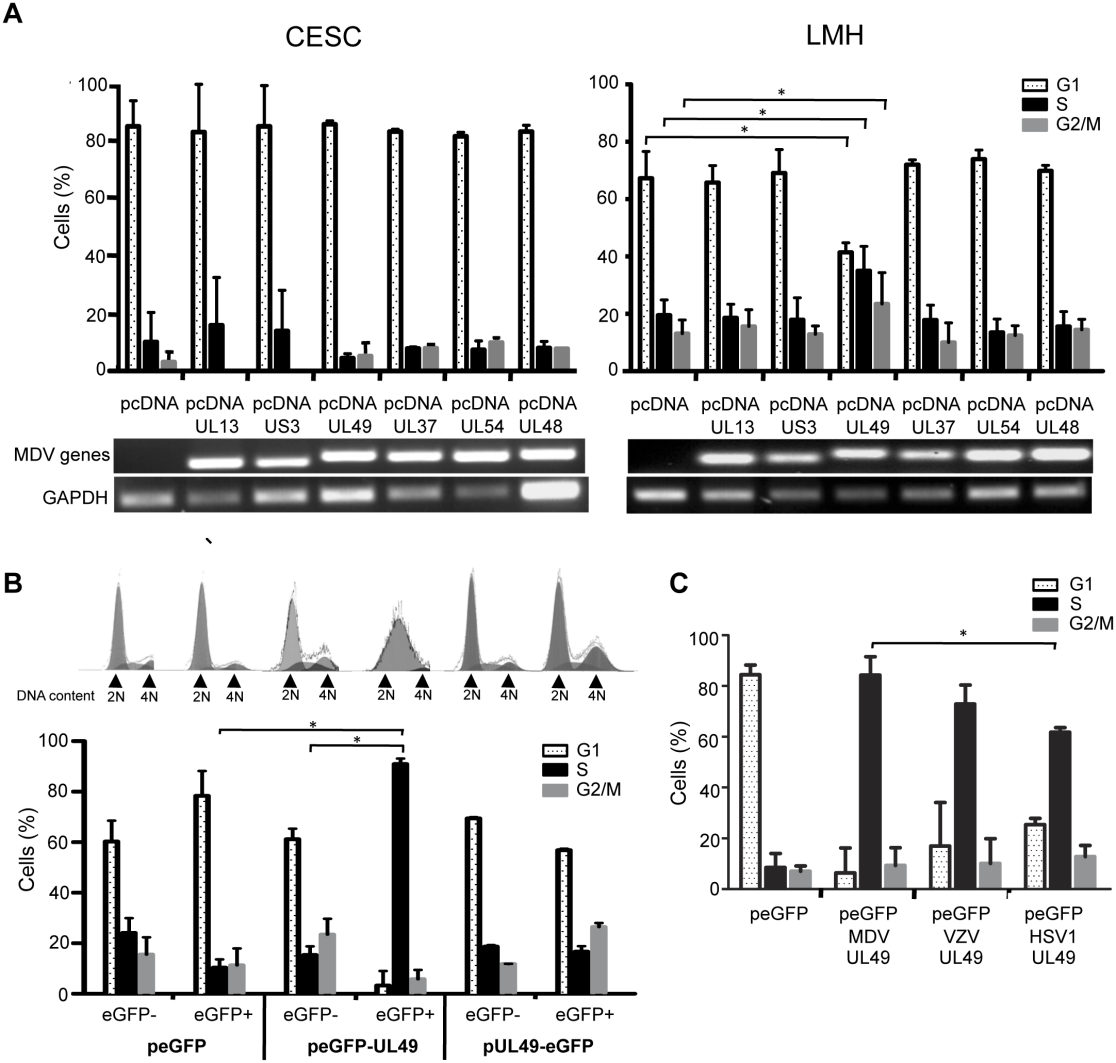
Identification of VP22 as a potent cell cycle modulator in proliferating cells. (A) Impact of the overexpression of different viral proteins on the cell cycle modulation. Primary (CESC) and high proliferating (LMH) cells were transfected with either an empty vector (pcDNA3) or with vectors expressing MDV-UL13, US3, UL49, UL37, UL54, or UL48 under CMV promoter control. Cell cycle analysis was performed at 48 h post-transfection by flow cytometry on the whole cell population (*p<0.05). The mRNA expression of the different MDV genes transfected was assayed by RT-PCR from total RNA extracted. GAPDH expression was analyzed for each sample as a control. The RT-PCR products were resolved by agarose (2%) gel electrophoresis. (B) Cell cycle regulation in VP22 expressing cells. LMH cells were transfected with plasmids harboring the UL49 gene in fusion with eGFP either at its N-terminal extremity (peGFP-UL49) or at its C-terminus (pUL49-eGFP) and with the empty vector peGFP. At 48 h post-transfection, cell cycle was analyzed specifically in UL49-expressing cells (eGFP+) and in non-transfected cells (eGFP−). Single parameter cytometry histograms are shown and the percentage of cells in the G1, S, and G2/M phases of the cell cycle is reported as bars. *p<0.05. (C) Cell cycle analysis in LMH cells expressing human VP22 orthologues. LMH cells were transfected with the empty vector peGFP and plasmids harboring the UL49 gene encoded by MDV, VZV and HSV-1 cloned in frame with eGFP at the N-terminus (peGFP-UL49). Cell cycle was analyzed at 48 h post-transfection exclusively in UL49-expressing cells (eGFP+) and the percentage of cells in the G1, S, and G2/M phases of the cell cycle is represented as bars. *p<0.05.

To identify viral factors involved in the regulation of the cell cycle during MDV infection, we tested the impact of the overexpression of six different viral proteins in CESC (low rate proliferating primary cells) and LMH cells (a cell line with high proliferative rate). Putative candidates were selected either on the basis of their biological activities that might influence host cell-encoded cell cycle regulators and/or on the basis of their essential role in the MDV life cycle. Because of the central role of cellular kinases in cell cycle progression, we were interested to test the two kinases encoded by MDV, pUL13 and pUS3. The ICP27 protein, encoded by the UL54 gene, was also included in the study as a multifunctional viral regulatory protein that has previously been shown to contribute to cell cycle modulation during HSV-1 infection [54], [55]. Three tegument proteins were also tested: the UL48-encoded viral trans-activator VP16, as well as pUL37 and VP22, both of which were shown to be essential for MDV growth (J-F Vautherot, unpublished data; [5]). Eukaryotic expression vectors harboring the viral candidate genes UL37, UL48, UL49, and UL54 (encoding pUL37, VP16, VP22 and ICP27, respectively) were transiently transfected into LMH or CESC cells. At 48 h post-transfection, the cell cycle status was analyzed as outlined earlier and the expression of each of the transfected MDV genes was verified by RT-PCR from total RNA extractions. No significant differences in the proportion of cells in each cell cycle phase was observed (Fig. 2A, left panel) for transfected CESC, suggesting that none of the overexpressed proteins was able to impact the cell cycle in quiescent CESC. In LMH cells, we also did not observe any cell cycle regulation in response to the expression of UL13, US3, UL37, UL54 and UL48, despite an effective expression of their respective mRNA (Fig. 2A lower panel). However, VP22 (pUL49) overexpression had a substantial effect on the cell cycle in the LMH cell line (Fig. 2A, right panel), as shown by the strong accumulation of cells in S-phase compared to control cells transfected with the empty vector pcDNA (35% versus 18% of cells in S-phase). Next, we tried to confirm our finding that VP22-expression alone results in an increase of cells in S-phase by transfecting LMH cells with plasmids encoding the VP22 protein fused to a eGFP-tag at its N- or C-terminus. Using an N-terminal eGFP-tagged VP22 protein (peGFP-UL49), we could confirm our finding that VP22 modulates the cell cycle, since more than 90% of LMH cells expressing VP22 (eGFP-positive cells) were blocked in S-phase (Fig. 2B). However, cells transfected with the plasmid encoding VP22 tagged at its C-terminus did not show any difference in cell cycle regulation compared to empty vector (peGFP)-transfected cells, which indicates that the location of the eGFP-tag at the carboxy-terminal extremity of the VP22 protein abrogates its activity on the cell cycle. Of note, the dramatic intra S-phase arrest observed with the N-terminal eGFP-tagged VP22 protein could be reproduced after overexpression of VP22 in two other avian cell lines: the chicken fibroblast cell line DF1 and the quail myoblast cell line QM7 (data not shown).

To verify whether the S-phase promoting activity of the MDV-encoded UL49 is conserved in other alphaherpesvirus orthologues, we tested the ability of VP22 encoded by HSV-1 and VZV to regulate the cell cycle. The HSV-1 and VZV-UL49 genes were cloned in-frame with eGFP and transiently overexpressed in the LMH cell line. At 48 hours post-transfection, the flow cytometry-based cell cycle analysis targeting transfected cells (eGFP-positive population) showed a significant S-phase arrest upon expression of all VP22 orthologues tested (Fig. 2C). VP22 orthologues derived from MDV and VZV proofed to be equally efficient, as approximately 80% of the cells expressing these VP22 were blocked in S-phase. Although HSV-1-VP22 substantially blocked the cell cycle progression in S-phase (61.8% of the transfected cells), it appeared slightly less efficient than other VP22 orthologues (especially MDV-VP22) in this process.

We thus identified a novel function for MDV-VP22 as a potent cell cycle modulator, with a strong S-phase promoting activity. We also revealed that an unmodified C-terminal extremity of VP22 is required for this process. Moreover this biological feature seems to be conserved among the human alphaherpesvirus, even though the two VP22 orthologues tested does not exhibit equal activity.

### Subcellular Localization of the VP22 Protein Encoded by MDV

**Figure 3:**
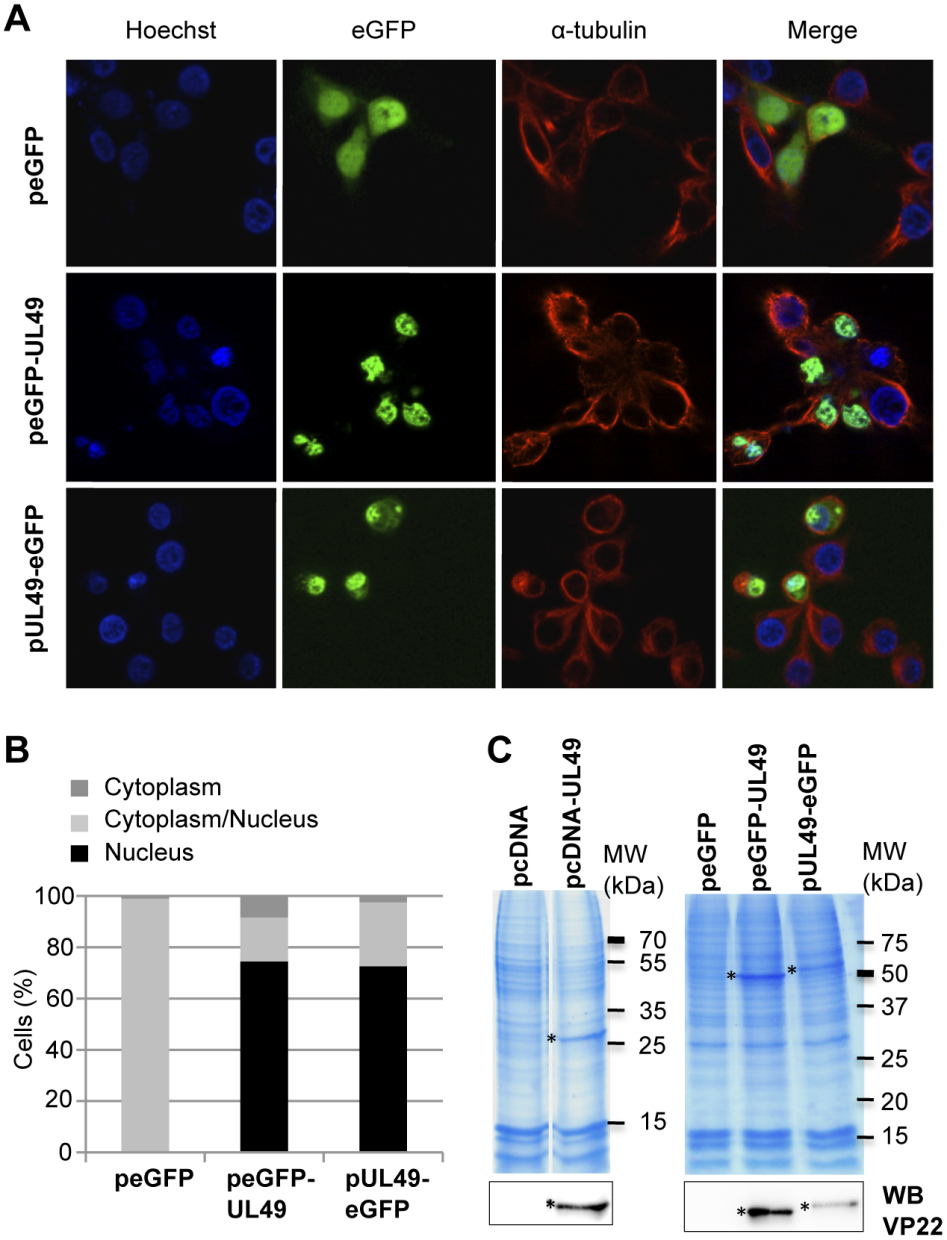
VP22 is predominantly located in the nucleus and associated to histones. (A–B) Subcellular localization of VP22 in LMH cells. The peGFP-UL49 and pUL49-eGFP plasmids as well as the peGFP empty vector were transfected in LMH. At 48 h post-transfection, cells were fixed with PFA 4% and subjected to immunofluorescence using anti-α-tubulin and AlexaFluor594-conjugated goat anti-rabbit IgG secondary antibody in order to demarcate the cytoplasm (red). Nuclei were counterstained with Hoechst 33342 (blue) and the eGFP proteins (green) were visualized directly by fluorescence microscopy. A representative example of the results obtained is shown (A). The nuclear/cytoplasm distribution of the eGFP proteins was estimated on an average of 100 cells and results are represented as stacked bars (B). (C) VP22 associates with histones. At 48 h post-transfection, histones were extracted in high salt conditions from LMH cells transfected with pcDNA, pcDNA3-UL49, peGFP, peGFP-UL49 or pUL49-eGFP. Extracts were separated by SDS-PAGE. Proteins were either directly stained in the gel with colloidal coomassie blue or transferred onto nitrocellulose membrane to perform a western blot analysis with an anti-MDV VP22 antibody (L13a). (*) indicates the presence of the VP22 proteins. The unmodified VP22 shows a molecular weight of 27 kDa and the N- or C-terminus tagged VP22 have a molecular weight of 55 kDa.

We took advantage of the differential cell cycle modulating activities of the C- or N-terminally eGFP-tagged VP22 fusion proteins to decipher which VP22 properties are crucial to mediate S-phase arrest. One hypothesis for different activity patterns could rest on differential subcellular distributions of the two proteins. To test this hypothesis, the two constructs peGFP-UL49 and pUL49-eGFP were transfected in LMH cells and the respective locations of the proteins were analyzed based on the eGFP signal by fluorescence microscopy at 48 h post transfection. In order to visualize more accurately the distribution of the two proteins, nuclei were stained with Hoechst 33342 and the cytoskeleton was stained with an anti-α-tubulin. Upon overexpression in LMH, the control eGFP protein (peGFP) was distributed all over the cells; the two VP22 proteins tagged at the N- or C-terminus did not show any significant difference in their cellular localization (Fig. 3A), with respectively 74,4% or 72,6% of eGFP-positive cells presenting an exclusive nuclear distribution and 17,2% or 25% showing a combined nuclear/cytoplasmic staining (Fig. 3B). Thus, the location of the eGFP tag, at the amino- or carboxy-terminus of VP22, does not seem to affect VP22 cellular distribution in LMH cells.

Another interesting feature of VP22 is its ability to bind to chromatin, especially to histones as it has previously been shown for the VP22 encoded by BoHV-1 [20], [56]. By performing a high-salt histones extraction protocol from cells transfected with either pcDNA-UL49 or pcDNA3.1 (empty vector), we found VP22 to be included in the histones fraction, as it is demonstrated by a 27 kDa band in the colloidal coomassie blue SDS-PAGE gel and by the VP22-specific antibody (L13a)-probed Western blot (shown in Fig. 3C-left panel). This result indicates that MDV-VP22 shares the ability of the VP22 encoded by BoHV-1 to interact with histones. In order to investigate the impact of the position of the eGFP tag on the ability of VP22 to associate with chromatin, we carried out a similar experiment using LMH cells transfected with peGFP, peGFP-UL49 or pUL49-eGFP. We observed that the VP22 tagged at its amino terminus could be co-extracted with histones and visualized as a specific 55 kDa band in coomassie blue-stained SDS-PAGE gel (Fig. 3C-right panel). However, the protein tagged at its carboxy terminus appeared to be significantly less retained in the histones fraction (Fig. 3C). These observations were confirmed by immunoblotting experiments using the anti-VP22 L13a antibody that show the presence of VP22 in the histone extracts prepared from cells expressing peGFP-UL49 and at a far lesser extent (about 4.5 fold) from the pUL49-eGFP transfected cells (Fig. 3C lower panel). All together, these data indicate that VP22 is predominantly targeted to the nucleus of LMH transfected cells independently of the eGFP tag location. However, the fusion of eGFP at the C-terminus of the VP22 protein affects its capacity to associate with chromatin.

### Accumulation of DNA Damages in VP22 Overexpressing Cells

**Figure 4:**
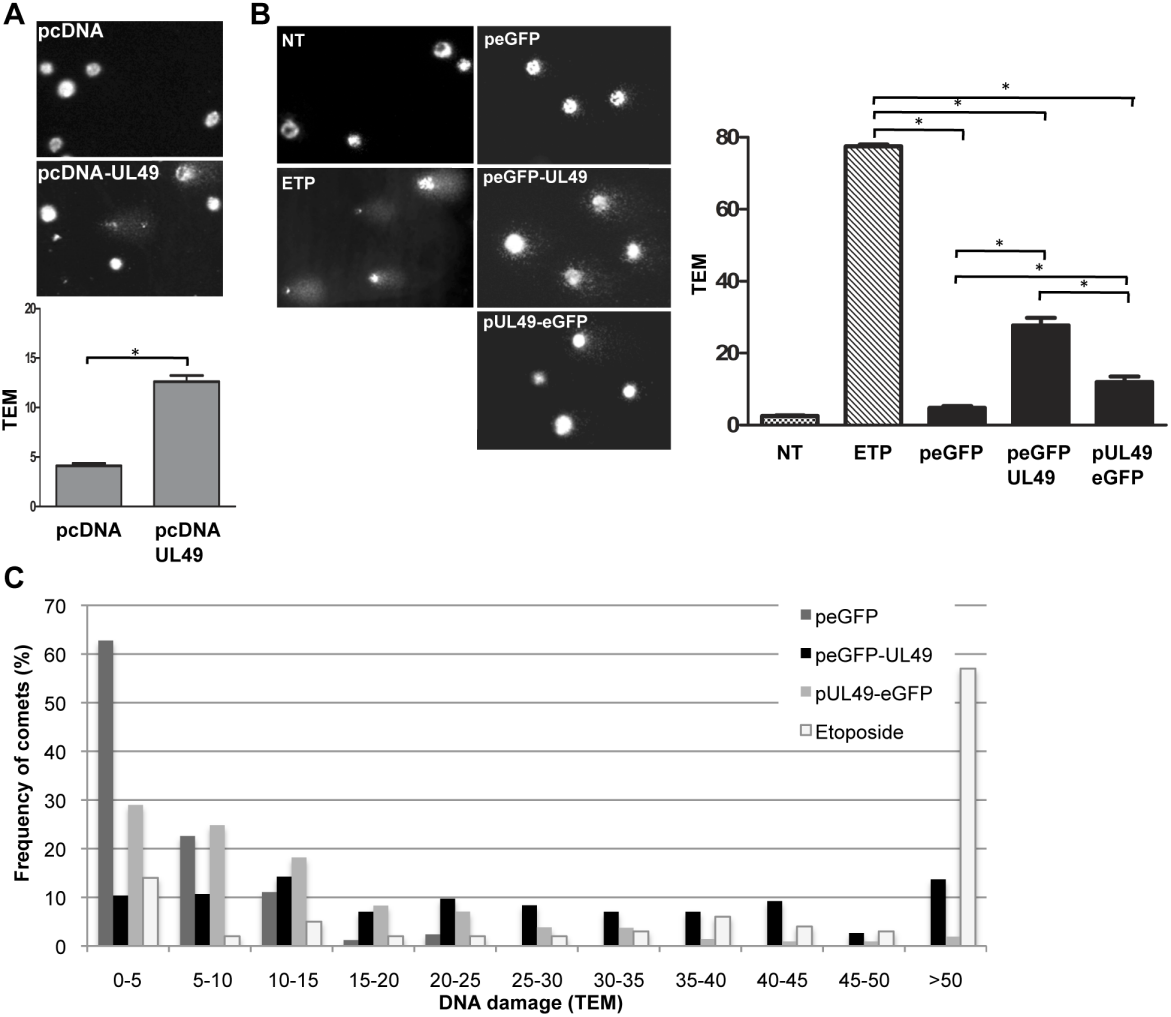
Expression of VP22 leads to cellular DNA damage. Detection of DNA damage in cells overexpressing VP22 by comet assay. (A–B) LMH cells were transfected with pcDNA, pcDNA3-UL49, peGFP, peGFP-UL49 or pUL49-eGFP as indicated. Non-treated (NT) LMH cells and cells treated with 1.5 µM etoposide (ETP) for 24 h were used as negative and positive control, respectively. After 24 h, the whole population of NT, ETP-treated cells, pcDNA and pcDNA3-UL49 transfected cells was directly subjected to comet assay. In the case of eGFP transfected cells, the EGFP positive cells were sorted by flow cytometry prior to comet assay analysis (B). Representative images of comets are shown as photographs. Quantitative and qualitative analyses of the comets are represented as histograms (± SD) on the basis of the calculation of the means of the tail extent moments (TEM) parameter measured with the CometScore software. (C) Frequency distribution of the comets with respect to their value of TEM. *p<0.05.

Arrest or delay in S phase can arise either from the occurrence of DNA damages, especially double strand breaks (DSB), or replication fork stalling [57], [58]. Since VP22 is able to drastically arrest the cell cycle in S phase and moreover seems to be associated to chromatin, we tested whether the overexpression of VP22 in LMH can induce DNA damages. LMH cells were transfected with pcDNA-UL49 or pcDNA3.1 (as a negative control) and DNA damages were analyzed by alkaline comet assay at 24 h post-transfection. This method, based on a single-cell gel electrophoresis, allows the detection of DNA breaks that are visualized as fragmented DNA exhibiting the shape of a comet’s tail. We could observe an increased number of comets in the population of cells transfected with VP22 compared to the cells transfected with the empty vector pcDNA3.1 (Fig. 4A). To estimate the extent of DNA damages, a more precise analysis with the Comet Score software was performed and the tail extent moment (TEM) was calculated. This parameter is calculated on the basis of the tail length, reflecting the severity of the damages and the amount of DNA in the tail relative to the head, which is an indicator of DNA break frequencies. The calculation of the TEM could show that cells expressing VP22 presented a significant higher TEM (12.62±0.62) than the cells transfected with pcDNA3.1 (4.11±0.25), indicating that the expression of VP22 seems to be associated with the occurrence of DNA damages in LMH cells (Fig. 4A lower panel). However, it should be stressed that this result reflects the DNA damage analysis on the whole population of pcDNA-UL49 transfected and non-transfected cells. Consequently to corroborate these findings and to determine whether the VP22 protein tagged at the C- or N-terminus was also able to induce DNA damage in LMH cells, we transfected the peGFP (empty vector), peGFP-UL49 or pUL49-eGFP plasmids in LMH cells and examined the onset of DNA damages by alkaline comet assay at 24 h post-transfection specifically in the eGFP positive cells sorted by flow cytometry. As positive control, LMH cells were treated with etoposide and as negative control, non-treated and non-transfected LMH cells were analyzed. We could readily observe comets from cells treated with etoposide and most of the cells overexpressing eGFP-UL49 and, to a lesser extent, from cells expressing the UL49-eGFP protein, whereas cells expressing peGFP produced almost no comets or comets with a shorter tail similar to the non-transfected cells (Fig. 4B). Calculation of TEM revealed that the mean tail moments of cells expressing eGFP-UL49 (27,73±2,11) or UL49-eGFP (11,99±1,52) is significantly higher than for cells transfected with peGFP (4,739±0,54), indicating that the expression of both tagged-VP22 proteins increases DNA damage in cells (Fig. 4B left panel). However, the damages were significantly more pronounced in cells expressing the protein tagged at its amino-terminal extremity than in cells expressing the C-terminally tagged version of VP22. Of note cells treated with etoposide showed a TEM of 77.47±0.55 thereby affirming the drastic induction of DNA damages by this DNA topoisomerase II inhibitor. In addition, we were interested to analyze the frequency distribution of tail moments (i.e. the percentage of cells presenting a defined TEM), which is representative for the number of cells encompassing damages (Fig. 4C). About 63% of cells transfected with peGFP had a tail moment inferior at 5, indicating that the majority of the cells contain non-damaged DNA or DNA with very limited damages. However, this cellular population decreased when VP22 was expressed both with the eGFP tag at the N-terminus or C-terminus (10% and 29%, respectively), and we could observe a marked increase of the proportion of cells presenting TEM values above 5 (89,6% and 71%, respectively). In particular, the expression of eGFP-UL49 tends to increase the frequency of cells with highly damaged DNA, more than 50% of the cells having a TEM>20 and 13,7% presenting TEM>50. In comparison, 19,7% of cells expressing UL49-eGFP showed a TEM>20 and only 1,9% a TEM>50. These observations indicate that the expression of VP22 in cells leads to an increased incidence DNA damaged cells and that damages are more severe when the VP22 is fused to eGFP at its N-terminal extremity. It should be however stressed that although the expression of VP22 leads to the occurrence of significant DNA damage, those damages are relatively less heavy than the ones induced by drugs such as etoposide that are responsible of potent damages (more than 55% of the comet having a TEM>50).

**Figure 5:**
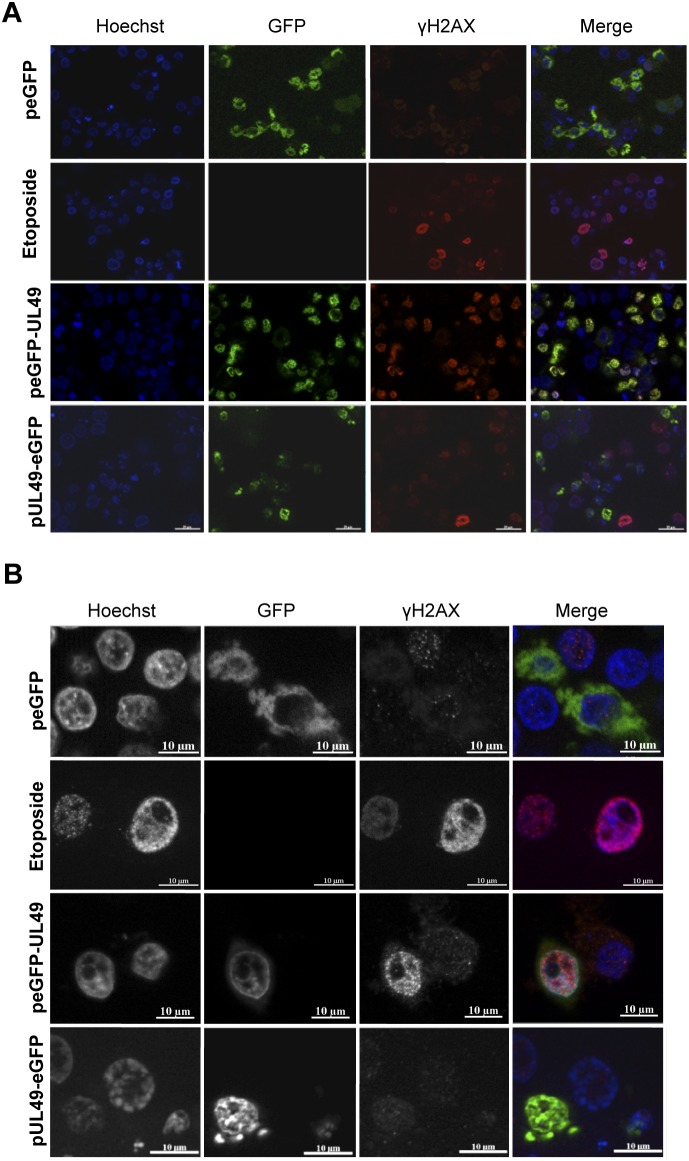
Accumulation of DNA double strand breaks in VP22 expressing cells. At 24-transfection, LMH cells expressing VP22 eGFP-tagged proteins (at N or C-terminus) or the empty vector peGFP were subjected to immunofluorescence using mouse anti-γH2AX and AlexaFluor594-conjugated goat anti-rabbit IgG antibodies (red). LMH cells exposed 24 h to etoposide were stained with the anti-γH2AX antibody and served as a positive control. Nuclei were stained with Hoechst 33342 (blue) and eGFP positive cells expressing VP22 were directly visualized by fluorescent microscopy (green) at low (A) and high magnifications (B).

In order to specify the nature of the DNA damages generated in cells expressing VP22, we monitored by immunofluorescence staining the expression and localization of γ-H2AX in LMH cells transfected with peGFP-UL49, pUL49-eGFP or with the empty vector peGFP. Because histone H2AX is rapidly phosphorylated (γ-H2AX) after generation of DNA double strand breaks (DSB), γ-H2AX is a preferential marker used to reveal these damages [59]. As positive control, the expression of γ-H2AX was also examined in cells exposed to etoposide [60]. We observed an overall increase of the staining intensity of the γ-H2AX DSB-marker in cells treated with etoposide and specifically in cells expressing eGFP-UL49 compared to non-transfected cells, peGFP transfected cells or UL49-eGFP expressing cells (Fig. 5A). Moreover, with higher magnification we could visualize that γ-H2AX formed discrete foci in the nucleus of eGFP-UL49 transfected cells as was also observed in etoposide-treated cells (Fig. 5B). This typical punctuated staining of γ-H2AX reflects its recruitment to sites of DNA damage and thus indicates that cells expressing VP22 tagged at its amino-terminus undergo multiple DSB.

## Discussion

In the present report, we show for the first time that MDV lytic infection leads to a dysregulation of the cell cycle progression of the host cell. MDV infection not only promotes the proliferation of primary embryonic skin cells, but also leads to an accumulation of infected cells in S-phase. This modulation of the cell cycle is accompanied by a significant up-regulation of cellular genes involved in G0/G1 transition (cyclin D, cdk6) and in G1 to S-phase progression (pRb, E2F1, c-myc, cyclins A). A substantial mRNA up-regulation of the cell cycle regulator p53 was also observed early after infection. Cell cycle modulation is a mechanism that is frequently exploited by viruses in order to facilitate viral replication. In contrast to small DNA viruses, a cellular S-phase environment is not mandatory for herpesviruses encoding their own DNA polymerase and accessory factors required for optimal viral replication [29]. Consequently, for most of alphaherpesviruses it has been demonstrated that they prevent the S-phase entry and rather activate the G1/S checkpoint. MDV is an alphaherpesvirus that shares a number of biological features with gammaherpesviruses, notably the viral lymphotropism and the ability to induce tumors. With respect to cell cycle modulation, our data suggest that MDV has adapted a similar strategy than EBV and KSHV, both of which were shown to promote cell cycle progression, especially into S-phase [33], [61]. It is conceivable that the MDV-mediated cell cycle modulation might also play a role in the multi-factorial events eventually leading to transformation and tumorigenesis. The S-phase is in fact the most vulnerable period of the cell cycle and a defect or inactivation of the key components of the intra S-phase checkpoint may predispose cells to oncogenic transformation (for review [57]).

The most unexpected discovery of our study is the identification of the MDV-VP22 protein as a potent trigger of cell cycle arrest in S-phase, as evidenced by the observation that its overexpression in proliferating LMH cells lead to the enrichment of up to 90% of transfected cells in S-phase. VP22 is a major component of the viral tegument of the *Alphaherpesvirinae*. While VP22 orthologs exhibit functional homology, their significance for alphaherpesviruses life cycle varies according to the virus species. This is well illustrated by previous studies showing that VP22 is dispensable for *in vitro* replication of PRV, HSV-1, and BoHV1, whereas it is essential for MDV and VZV replication [5], [7], [8], [10], [11], [12]. However, the biological properties of VP22 that determine its key role in the life cycle of MDV remain still unknown. One hypothesis is based on the crucial function of VP22 in cell-to-cell spread [5], [18]. We can also not exclude that a rapid distribution of VP22 after viral entry might prepare an optimal environment for viral replication by inducing an S-phase arrest.

Several viral proteins encoded by herpesviruses have been shown to have an impact on the cell cycle. Among the ones encoded by the *Alphaherpesvirinae*, the ICP0 protein is probably the best studied. This multifunctional protein required for efficient HSV-1 lytic replication and reactivation from latency, has been identified as a major cell cycle modulator that is able to act either on the G1/S or at the G2/M checkpoints [62]. However, the observation that ICP0 deficient mutant viruses are still capable to elicit proliferation arrest indicates that other viral factors also impact the cell cycle [62]. Notably, the immediate early protein ICP27 was shown to be essential for the G1/S cell cycle arrest triggered by HSV-1, with ICP4, ICP0, and the virion host shutoff protein acting as contributors [55]. Hence, MDV and HSV-1 appear to employ differential cell cycle modulation mechanisms as MDV does not encode a functional ICP0 protein and we could not detect any effect of ICP27-overexpression on cell proliferation. It is an interesting speculation that MDV may have evolved a distinct mechanism for cell cycle modulation that crucially involves VP22 in order to compensate for the absence of ICP0 activities. It should be noted that the overexpression of the VP22 proteins encoded by HSV-1 or VZV also resulted in a dramatic arrest of the cell cycle in S-phase in transfected LMH cells. This finding suggests that VP22 might also contribute to the modulation of the cell cycle in the context of infections with human herpesviruses. While screening for viral factors that are involved in the MDV-associated cell cycle regulation, we also tested whether the activity of the two MDV-encoded serine-threonine kinases pUS3 and pUL13 could have a cell cycle regulatory effect. Indeed, it is well known that cell cycle progression is submitted to a tight regulation mediated by kinases and phosphatases. Overexpression of UL13 and/or US3 in low proliferating cells (CESC) or high proliferating cells (LMH) had no effect on the cell cycle, thus excluding a direct involvement of these kinases in the cell cycle modulation. However, pUS3 and pUL13 are able to phosphorylate various cellular and viral proteins, including the VP22 proteins encoded by HSV-1 and -2, as well as BoHV-1 [63], [64], [65]. So far, the phosphorylation status of MDV-VP22 during infection has not been investigated, and we cannot exclude post-translational modifications of MDV-VP22 by UL13 and/or US3, as previously shown for other alphaherpesviruses.

Intra-S checkpoints activation mainly reflects DNA breaks or stalled replication fork formation [58]. In order to identify the molecular mechanisms underlying the VP22-driven S-phase arrest, we focused on the impact of VP22 expression on the generation of DNA damage in the host cell genome. Following overexpression of VP22 in proliferating cells, we could indeed show by comet assay that the presence of VP22 coincided with the occurrence of massive DNA damage. Moreover, VP22-expressing cells showed an increased staining of the phosphorylated form of H2AX, suggesting that the DNA lesions observed are double strand breaks [59]. Interestingly, the VP22-mediated generation of DNA damages seems to be tightly associated to the cell cycle modulation property of VP22. This was evidenced by our comparative experimental approach using two versions of the VP22 protein tagged either at its N- or C-terminus. The data from this experiment show that virtually all cells expressing eGFP-VP22 (N-terminal eGFP-tag) are arrested in S-phase and present severe DNA damage, whereas cells expressing VP22-eGFP (C-terminal eGFP-tag) are not affected in their cell cycle progression and show significantly less DNA lesions. Of note, all our attempts to generate a LMH stable cell line overexpressing the MDV-VP22 protein failed due to a high level of cellular mortality. These observations raised the question of the potential toxicity of VP22, which might find an explanation in the induction of double strand breaks in cells overexpressing VP22.

The mechanisms by which VP22 induces S-phase arrest and DNA breaks still remain to be elucidated. However, among the characteristics of VP22, we can speculate that its capacity to interact with chromatin and histones might participate to those processes. Interactions of VP22 protein with nucleosomes were previously demonstrated for the BoHV-1-encoded VP22, which physically interacts with nucleosome-associated histones and thereby causes an impaired acetylation of histone H4 [21], [56]. In addition, for MDV-VP22, the regions allowing interaction with heterochromatin were previously defined [17]. In the present study, we confirmed that MDV-VP22 is found predominately in the nucleus of cells following overexpression in LMH cells. We also found that an N-terminally eGFP-tagged MDV-VP22 can be extracted from chromatin preparations together with histones. However, for a C-terminally eGFP-tagged MDV-VP22, the efficiency of recovery from histones extracts was far less, suggesting that an unmodified C-terminal extremity of VP22 is necessary for the association of VP22 with chromatin. Together, these observations suggest that the abilities of VP22 to arrest the cell cycle in S-phase and to effect DNA damage are linked to its direct or indirect interaction with histones and/or chromatin. According to this model, it is conceivable that the interaction of VP22 with chromatin or histones may disturb the unwinding of DNA in a similar fashion than cellular helicases or topoisomerases by preventing access to the DNA replication machinery. Alternatively, it can be speculated that an association of VP22 with DNA/histones may cause physical tension of the DNA double helix eventually leading to DNA breaks.

Activation of DNA damage response (DDR) pathways is well documented for a number of viruses, especially tumorigenic viruses and plays a central role in viral replication [46], [47]. While our data only provide clear evidence for a role of VP22 as a powerful inducer of DNA damage in a non-infectious context, it can be assumed that DDR activation might play a role in MDV replication and/or MD pathogenesis. Due to the critical role of VP22 for MDV-replication [5], it is so far impossible to evidence the function of VP22 as a major cell cycle regulatory factor during MDV infection by using a VP22-deleted virus. However, Jarosinsky *et al*. have demonstrated that a recombinant MDV harboring a VP22 protein tagged with eGFP at the C-terminus (a construct that is identical to the VP22-eGFP used in the present study) showed a drastic decrease in its ability to induce MD in infected chickens, with only 10% of the chicken developing tumors [19]. In addition, we have recently observed that a recombinant virus with the VP22 protein tagged at the N-terminus is also attenuated, but in a lower extent, with 33 to 66% of the infected chickens developing MD lymphoma [6]. Although the impairment of pathogenicity of the recombinant MDV studied by Jarosinski *et al*. could in parts be explained by a lower viral replication efficiency *in vivo*, in view of our data, it can also be speculated that the defect in tumor development observed by the authors might be due to the loss of the ability of the C-terminally tagged VP22 protein to induce S-phase arrest and DNA damage.

In conclusion, our findings provide new insights into herpesvirus-cell host interactions by demonstrating that the oncogenic alphaherpesvirus MDV affects the cell cycle progression in infected cells. Moreover, we could assign a novel role to the VP22 tegument protein as a potent cell cycle modulator, property that seems to be associated to its ability to induce DNA damages in cells. Current efforts are under way to elucidate the detailed mechanisms of VP22-induced DNA damage response, and its role during viral infection, especially with respect to a possible involvement of DDR in MDV replication and/or the establishment of MDV-latency and subsequent lymphoma formation.
